# “How?” and “Why?”

**Published:** 1897-06

**Authors:** 


### "How?" and "Why?"

In his address before the British Medical Association, Sir Wm. Broadbent says: "Morphia suspends the activity of the nerve centers. But how? What chemical or molecular change takes place in the tissues? How is it that the slightest change in the composition of the morphia molecule radically alters its effects? The physician can not tell. The salts of potassium and of sodium are almost exactly similar. Yet a minute quantity of the former injected into a vein will paralyze the heart and destroy life, while the latter may be turned into the circulation wholesale with no bad result. Why is it? Why is so simple a substance as prussic acid so deadly a poison? A thousand of such questions may be asked. None of them can yet be answered. We know that these things do thus and so. How they do it we do not know; but until we do medicine will scarcely become an exact science. That we shall one day attain such knowledge is confidently to be expected. That must be the work of chemistry; and when we remember that the science of chemistry is scarcely more than a century old, and when we consider the bewildering scope and importance of its achievements in that century, it is surely not too much to hope great things from its future labors." —*Mining and Scientific Press*, Nov. 23, 1895.

